# Community interactions among microbes give rise to host-microbiome mutualisms in an aquatic plant

**DOI:** 10.1128/mbio.00972-24

**Published:** 2024-06-21

**Authors:** Jason R. Laurich, Emma Lash, Anna M. O'Brien, Oxana Pogoutse, Megan E. Frederickson

**Affiliations:** 1 Department of Ecology & Evolutionary Biology, University of Toronto, Toronto, Ontario, Canada; 2 Department of Molecular, Cellular, and Biomedical Sciences, University of New Hampshire, Durham, New Hampshire, USA; Corporación CorpoGen, Bogotá D.C., Colombia

**Keywords:** microbiome, mutualism, competition, Lemna minor, plant-microbe interactions

## Abstract

Microbiomes often benefit plants, conferring resistance to pathogens, improving stress tolerance, or promoting plant growth. As potential plant mutualists, however, microbiomes are not a single organism but a community of species with complex interactions among microbial taxa and between microbes and their shared host. The nature of ecological interactions among microbes in the microbiome can have important consequences for the net effects of microbiomes on hosts. Here, we compared the effects of individual microbial strains and 10-strain synthetic communities on microbial productivity and host growth using the common duckweed *Lemna minor* and a synthetic, simplified version of its native microbiome. Except for *Pseudomonas protegens*, which was a mutualist when tested alone, all of the single strains we tested were commensals on hosts, benefiting from plant presence but not increasing host growth relative to uninoculated controls. However, 10-strain synthetic microbial communities increased both microbial productivity and duckweed growth more than the average single-strain inoculation and uninoculated controls, meaning that host-microbiome mutualisms can emerge from community interactions among microbes on hosts. The effects of community inoculation were sub-additive, suggesting at least some competition among microbes in the duckweed microbiome. We also investigated the relationship between *L. minor* fitness and that of its microbes, providing some of the first empirical estimates of broad fitness alignment between plants and members of their microbiomes; hosts grew faster with more productive microbes or microbiomes.

**IMPORTANCE:**

There is currently substantial interest in engineering synthetic microbiomes for health or agricultural applications. One key question is how multi-strain microbial communities differ from single microbial strains in their productivity and effects on hosts. We tested 20 single bacterial strains and 2 distinct 10-strain synthetic communities on plant hosts and found that 10-strain communities led to faster host growth and greater microbial productivity than the average, but not the best, single strain. Furthermore, the microbial strains or communities that achieved the greatest cell densities were also the most beneficial to their hosts, showing that both specific single strains and multi-strain synthetic communities can engage in high-quality mutualisms with their hosts. Our results suggest that ~5% of single strains, as well as multi-strain synthetic communities comprised largely of commensal microbes, can benefit hosts and result in effective host-microbe mutualisms.

## INTRODUCTION

Plants and animals harbor diverse microbiota that often affect the phenotypes or fitness of their hosts. The microbes in these microbiomes interact with one another, just like plants and animals do in more familiar ecosystems such as grasslands or rainforests. Microbes living together in or on hosts can compete for resources, exploit other microbes in interactions akin to predation or parasitism, or cooperate in mutualisms. However, the relative importance of these interaction types (i.e., competition, exploitation, or mutualism) in microbial communities is the subject of debate (1), and microbiome science is only beginning to interrogate the consequences of microbial interactions within the microbiome for their combined effects on hosts (2, 3).

Several studies have assessed the frequency of competition, exploitation, and mutualism among microbes in communities (1, 4–7). One tested many possible combinations of 72 bacteria isolated from rainwater pools in tree holes and found that most bacteria grow better alone than with other microbes (4), suggesting that competition prevails among culturable bacteria. However, when Kehe et al. (6) used an ultrahigh-throughput platform to test over 180,000 combinations of 20 soil bacteria, they found more positive interactions than expected; about 40% of bacteria grew better with other bacteria than alone, although most of these interactions were exploitative, not mutualistic. Recently, Palmer and Foster (1) synthesized the available evidence from multiple studies of microbial communities and concluded that “negative interactions prevail, and cooperation, where both species benefit, is typically rare.”

Competition among microbes in the microbiome may benefit hosts, if host pathogens are competitively suppressed or excluded by other microbes (3, 8). Pathogen suppression is a primary benefit of plant microbiomes that is often mediated by a small subset of strains (3, 9–12). If pathogen suppression emerges as a consequence of competition among strains, then internal antagonism in microbiomes has the potential to result in overall microbiome cooperation with hosts. Such dynamics could also explain why more diverse microbiomes can provide greater pathogen suppression benefits to their hosts (9). Consistent with ecological theory, more diverse microbiomes may be more likely to contain strains that suppress pathogens through competitive dominance (e.g., the sampling effect [13]). This raises the possibility that mutualistic outcomes between certain pathogen-suppressing microbes and hosts may be more visible with increasing microbiome complexity, precisely because more diverse communities are more likely to contain the very pathogens whose inhibition demonstrates their positive effects. More generally, diversity within the microbiome can increase the overall productivity and ecosystem services provided by plant microbes if diverse microbiomes are more likely to contain “keystone” microbes that have disproportionately large direct effects on host phenotypes or strongly shape microbial community composition (9, 12, 14–19).

However, not all interactions among microbes are competitive, and microbial versions of the classic biodiversity-ecosystem function (BEF) relationship often documented for plant communities (9, 20–24) may also arise through facilitation among microbes (2). Many bacterial metabolites are leaky, diffusing across cell membranes into community space. This can lead to the evolution of cross-feeding or nutritional dependence among bacteria, thereby increasing productivity in more diverse microbial communities (25–27). Furthermore, indirect ecosystem services provided by microbiomes, such as toxin or antibiotic degradation or biofilm formation, can be strongly selected for in some community members through market-like dynamics that redound to the benefit of all strains (28, 29). In host-microbiome interactions, the evolution of niche complementarity favored by these processes and the production of functionally distinct host rewards produced by community members are further expected to synergistically increase benefits to hosts (30, 31).

Microbial cooperation may be more likely in the presence of a host, which has the potential to change the nature of microbe-microbe interactions within a community. Even if microbes compete for resources, if one microbe promotes host growth in a way that generates increased supply of host rewards to the microbiome as a whole, then other microbes will benefit from its presence. Plant-derived organic carbon is likely one such shared reward for microbes; plants secrete up to 44% of their fixed carbon as root exudates (32), resulting in microbial densities in the rhizosphere far in excess of microbial densities in surrounding environments (33). Few of the studies highlighted by Palmer and Foster (1) grew microbes in association with a host (but see references 5, 7) and even fewer simultaneously compared single and multiple strains of microbes in terms of their effects on both microbial and host growth.

Whether microbes benefit one another indirectly by promoting the growth of their shared host depends on what kind of benefits microbes confer to hosts and whether those benefits feed back to all microbes living on a host (i.e., as a “public good”) or to only one or a few strains. In plants, in addition to suppressing pathogens (9–11, 34), microbes can confer resilience against environmental stressors such as elevated salinity (35, 36), drought (37), or flooding (38) or even degrade or detoxify detrimental pollutants such as chromium, arsenic, or phenols (15, 17, 39). Plant microbiomes can also promote plant growth by fixing nitrogen (40, 41), solubilizing phosphates (42), or producing plant growth-promoting hormones or compounds such as indoles and auxins (19, 43). However, whether these benefits of microbes to hosts feed back to benefit the microbes themselves remains an open empirical question in most systems, because few studies measure the benefits of host association to microbes or the extent of fitness alignment or conflict between host and microbial partners (44, 45). Fitness feedbacks between hosts and symbionts determine how cooperation evolves between species (46), and the evolution of genuinely mutualistic interactions between plants and microbes is no less dependent on such factors than other relationships (47, 48).

The only plant-microbe interaction in which fitness alignment or conflict has received substantial attention is the legume-rhizobium mutualism, in which legumes host rhizobacteria in root nodules where they exchange fixed carbon for fixed nitrogen. Inoculations of rhizobia strains onto legumes have revealed mostly positive fitness correlations between partners (49, 50), implying that natural selection generally favors the evolution of more beneficial rhizobia. Indeed, recent evolution experiments with rhizobia have directly observed the evolution of greater host benefits in real time (51). In contrast, whether plant-microbe fitness correlations are positive, negative, or neutral in other systems is a largely open question (but see reference 52), meaning we have a limited understanding of whether selection favors more or less beneficial microbes in symbioses beyond legumes and rhizobia. That plants often benefit substantially from their microbiomes is well documented (e.g., references 34, 36, 37), and many plants invest heavily in the sort of reciprocal exchange of nutrients that fuel mutualistic interactions through rewards such as root exudates (32). However, we would also expect to find that some microbes in plant microbiomes are pathogenic and proliferate rapidly by over-exploiting plants and reducing plant fitness (18). Furthermore, fitness correlations measured in legumes and rhizobia generally involve comparing the performance of many closely related rhizobia strains on hosts (i.e., phenotyping many isolates of the same rhizobium species), while plant microbiomes are highly diverse with many microbial lineages competing for host rewards. Whether natural selection favors the most beneficial microbes in diverse plant microbiomes or whether microbial fitness is largely uncoupled from plant benefits deserves greater empirical attention in plant-microbiome interactions.

Here, we leveraged the relationship between the common duckweed *Lemna minor* and its microbiome to investigate several fundamental questions pertaining to the ecology and evolution of plant-microbiome interactions. Duckweeds (Lemnaceae) are the world’s fastest growing and smallest angiosperms (53). Their rapid growth rates, coupled with their nearly entirely clonal reproduction through the budding of fronds (54, 55), facilitates measurements of host fitness at high replication in a laboratory setting (56). The duckweed microbiome resembles that of terrestrial angiosperms (57) and strains in the families Aeromonadaceae, Caulobacteraceae, Chitinophagaceae, Comamonadaceae, Enterobacteriaceae, Flavobacteriaceae, Pseudomonadaceae, Rhizobiaceae, Rhodospirillaceae, and Sphingomonadaceae are common members of the core duckweed microbiome (24, 52, 57–60). Previous research has characterized the effects of whole microbiome inoculation on duckweeds (e.g., references 52, 61). In this study, we compared the effects of single microbial strains and 10-strain synthetic microbial communities inoculated onto sterilized *L. minor* plants to investigate the effects of microbiome community interactions on microbial productivity (4–6) and host fitness and quantified the degree of fitness alignment between *L. minor* and its microbes. Specifically, we sought to address the following questions. (i) How do interactions among microbes affect microbial productivity in the host versus free-living environment? (ii) How do microbiome diversity and microbe-microbe interactions affect the benefits microbiomes provide to their hosts? And (iii) how aligned are the fitness interests of *L. minor* and its microbes?

## MATERIALS AND METHODS

### Collection and culturing of *Lemna minor* and its microbiome

*Lemna minor* is a small, floating, aquatic macrophyte with a worldwide distribution in temperate zones. Like other duckweeds, *L. minor* exhibits a highly reduced morphology and simple life history (53). Plants consist of 1–4 fronds ranging in length from 1 to 8 mm, with a single adventitious root per frond (54). While *L. minor* is capable of sexual reproduction, flowering is extremely rare in *L. minor* (62), and populations have little segregating genetic diversity due to high rates of clonal reproduction through frond budding (54, 55, 63). *Lemna minor* boasts an extremely high growth rate, doubling approximately every 4 days (64) and can establish dense, dominant vegetative mats in ponds and other slow-moving bodies of water (65, 66). Distinguishing *L. minor* from other closely related duckweeds can be difficult as a result of their highly simplified morphology, and genetic markers suggest that plants identified as *L. minor* may sometimes be *L*. × *japonica*, a hybrid of *L. minor* and *Lemna turionifera* (67, 68). Nonetheless, in keeping with previous studies (52, 58), we refer to the duckweeds used in these experiments as *L. minor*.

**Fig 1:**
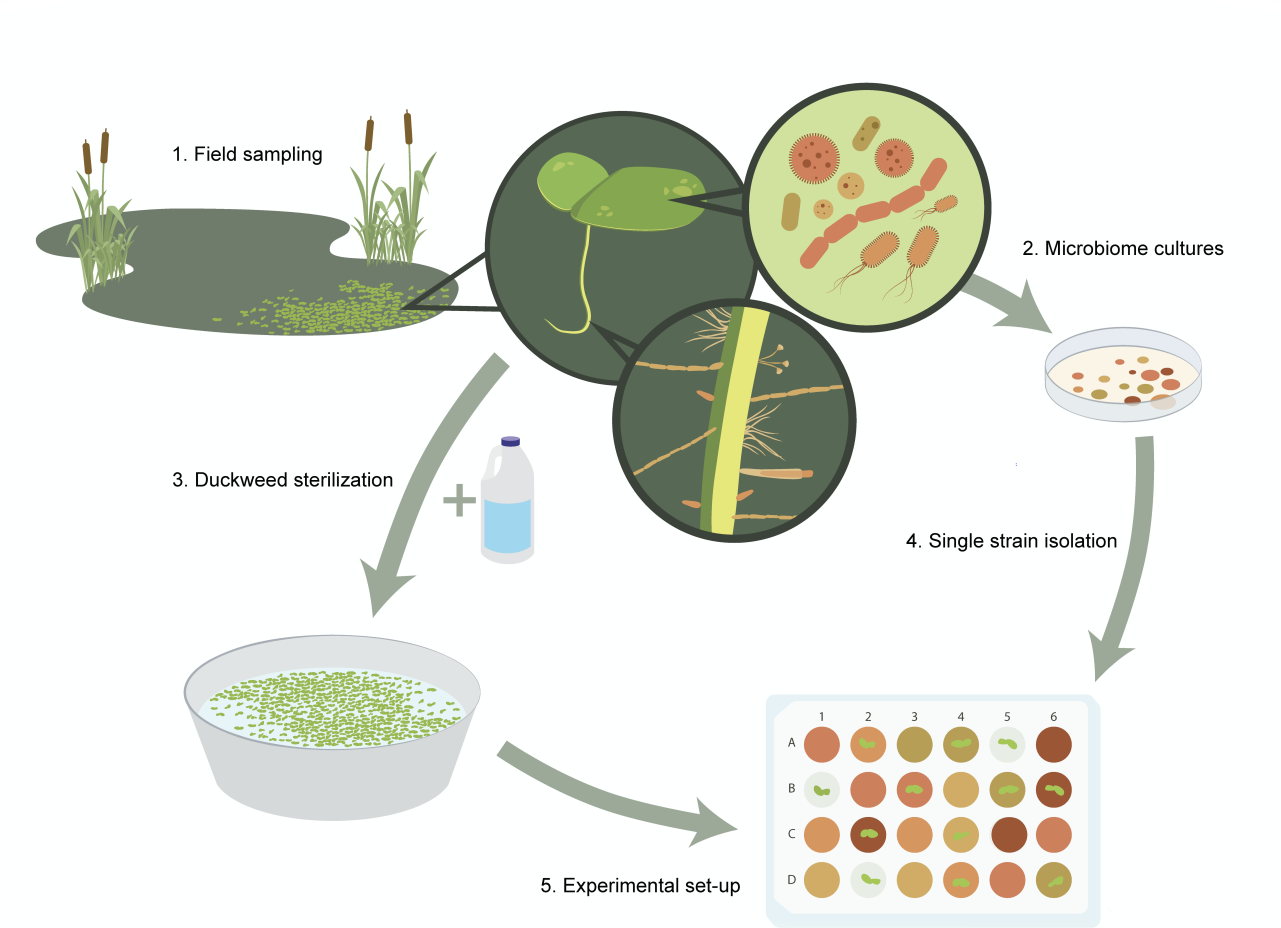
Graphic showing field sampling of *Lemna minor* and its associated microbiome (1), microbiome culturing (2), bleach sterilization of duckweeds (3), isolation of single bacterial strains (4), and set-up of duckweed-bacteria experiments in 24-well plates (5), with colors representing distinct microbe treatments. Graphic by Jacelyn Shu (https://www.jacelyndesigns.com/).

We collected *L. minor* plants from two locations in the Greater Toronto area in the summer of 2017: Churchill Marsh (43.77°N, 80.02°W) and Wellspring Pond (43.48°N, 79.72°W). Immediately after collecting live plants in the field, we cultured components of the *Lemna minor* microbiome by crushing the tissue of approximately six fronds per population and streaking this mixture onto yeast mannitol agar plates (Fig. 1). We used a swab to apply microbes from this crushed tissue to our plates and repeatedly streaked diminishing volumes across plates using a flame-sterilized bacterial spreader to reduce the density of bacterial colonies. We cultured plates at 29°C for 5 days to generate a “master plate” containing a diversity of culturable strains present in the duckweed microbiome. These plates capture only a subset of the bacterial strains present in the duckweed microbiome, displaying an order of magnitude less diversity than plants in the field (52, 58). The microbes we cultured are biased toward epiphytic bacterial strains and, as in other microbiome studies, under-represent the many important bacterial endophytes, fungi, protists, and viruses that interact with *L. minor* in nature (19, 43, 52, 57, 58, 69–71). Nevertheless, they represent many of the most abundant taxa present in the *L. minor* microbiome (52, 58), and many of these bacteria affect duckweed growth and phenotypes (15, 19, 40, 42, 52, 58).

Where past experiments focused on the effects of whole community inoculation (52, 61), here, we focused on interactions between duckweeds and individual strains isolated from their microbiome, in addition to simplified, 10-strain synthetic microbiomes. We isolated single bacterial strains from master plates by serially re-streaking colonies with a flame-sterilized bacterial spreader and plating colonies until the colonies had a single phenotype (Fig. 1). We selected bacterial colonies for isolation and cultivation on the basis of their morphology (colony color, size, and shape) to generate as much diversity in our experimental strains as possible. We then grew liquid cultures of individual bacteria by inoculating liquid yeast mannitol media with cells taken from a single colony. We placed these liquid cultures in a shaking incubator at 29°C for 5 days to generate experimental inocula. We then used a NovoCyte Flow Cytometer (ACEA Biosciences Inc., San Diego, CA, USA) to measure the concentration of bacterial cultures in liquid media before diluting to 10^5^ cells per 50 µL of inoculum.

To generate isoclonal lines of *L. minor*, we transferred duckweed fronds from each population to Krazčič growth media (72) in 500 mL Mason jars. We maintained stock cultures in an environmental chamber set to cycle between a 16-hour period at 23°C and 150 *μ*mol/m^2^ light followed by 8 hours of darkness at 18°C. We then placed individual fronds in sterile media; all plants used in subsequent analyses and experiments are the clonal descendants of a single frond per population. Once our isogenic cultures reached high density, we sterilized duckweed fronds by vortexing plants twice in phosphate-buffered saline for 5 minutes before immersing them in 1% sodium hypochlorite bleach for 1 minute (Fig. 1). We then thoroughly rinsed bleached fronds in autoclaved distilled water and transferred plants to sterile growth media. This sterilization process disrupts and simplifies the natural microbiome of *L. minor* and is particularly effective at sterilizing epiphytic bacteria associated with duckweed fronds and roots but does not always entirely remove endophytic species (19, 43). We tested the effective sterility of *L. minor* fronds roughly 1 week later by placing them in growth media enriched with 5 g/L sucrose and 1 g/L yeast extract.

### Sequencing of field microbiomes and individual bacterial strains

We used 16S rRNA amplicon sequencing to characterize the bacterial communities associated with *Lemna minor* from the same 2017 Churchill Marsh and Wellspring Pond field samples that generated our single-strain isolates. We extracted DNA from field-collected frozen duckweed tissue with DNeasy PowerSoil Kits (Qiagen), using the recommended fresh tissue weight (0.25 g). We then sent 10 ng of DNA to Génome Québec for PCR amplification of 16S rDNA at the V3–V4 region with primers 341f/805r, library preparation, and barcoded, paired-end, 250-bp format sequencing on an Illumina MiSeq System. We profiled the resulting reads using QIIME 2 (73) to clean, trim, process, and taxonomically identify amplicon sequence variants (ASVs). We then inserted ASVs into the Greengenes2 reference phylogenetic tree (74).

We extracted bacterial DNA from liquid cultures of each single-strain isolate using a Polyzyme digestion protocol (75). We digested cells in MetaPolyzyme Multilytic Enzyme Mix (Sigma) and lysed cells in Puregene Cell Lysis solution (Qiagen). After extracting DNA, we performed PCR reactions using universal bacterial 16S rDNA primers (1492R, 5-TAC GGY TAC CTT GTT ACG ACT T-3, and 27F, 5-AGA GTT TGA TCM TGG CTC AG-3) (76). We then purified our PCR products with a QIAquick PCR Purification Kit (Qiagen) and sent purified DNA to either the Centre for the Analysis of Genome Evolution and Function at the University of Toronto or the Centre for Applied Genomics at Toronto’s Hospital for Sick Children for Sanger sequencing on an Applied Biosystems 3730 DNA Analyzer using standard protocols.

We used Geneious Prime 2024.0.3 to trim and merge forward and reverse sequences and identified our strains using the blastn algorithm against the NCBI 16S ribosomal RNA database for Bacteria and Archaea (77). We regarded matches with greater than 98% sequence similarity as the same species and matches with greater than 95% sequence similarity as the same genus. Five strains did not match any NCBI 16S rRNA sequence at greater than 95% sequence identity (Tables S1 and S2). Two of these five strains were at least 96% similar in sequence identity to ASVs identified by the QIIME 2 pipeline (see above) as belonging to the genus *Allorhizobium*, so we called them *Allorhizobium* sp. 1 and *Allorhizobium* sp. 2. The other three strains shared less than 95% of their sequence with any field ASV, so we refer to them as unidentified strains in their respective bacterial family or order. We used the MAFFT and FastTree algorithms in QIIME 2 to align the full-length 16S rRNA sequences for the 20 single-strain isolates with the V3–V4 region ASVs for Churchill and Wellspring field samples and build a phylogenetic tree, which we visualized with the R package ggtree (78).

### Experimental design and procedures

We inoculated sterilized *Lemna minor* plants from both field sites with individual bacterial strains and 10-strain synthetic bacterial communities (Fig. 1). We measured how individual bacterial strains and synthetic communities (20 replicates per treatment) affected both microbial and host growth. For each population, plants were inoculated with microbes cultured from field-collected plants from that same population, i.e., we inoculated Churchill duckweeds with Churchill microbes and Wellspring duckweeds with Wellspring microbes. Thus, the plant-microbe associations may be locally adapted or co-adapted (61).

We filled 24-well plates with autoclaved, sterile culture media. After rinsing to remove all traces of enriched media from sterilized plants, we randomly assigned plants (1–3 fronds) to wells, leaving some wells empty for the no-host treatment. We then sealed plates with a gas-permeable membrane (Breathe Easier, Sigma-Aldrich). We inoculated each well with 50 µL of a randomly assigned bacterial or control inoculum by pipetting through the gas-permeable membrane and re-sealed plates with a second membrane upon completion (Breathe Easy, Sigma-Aldrich). The control inoculum consisted of autoclaved culture media, while inocula for single bacterial strain treatments contained approx. 10^5^ cells and the synthetic microbiome inoculum contained approx. 10^4^ cells from each of 10 single bacterial cultures (resulting in a 10-strain community with the same final cell concentration as single-strain inocula). We included no-host treatments in which we inoculated wells with bacteria but did not add plants, to compare bacterial growth in the presence and absence of hosts.

After loading wells with plants, bacteria, or both, we took images of the plates and placed them in an environmental chamber (16:8 h light/dark, 23/18°C cycle) for 10 days. We selected this 10-day period as it is consistent with other work on duckweeds (e.g., references 52, 58) and prevents the overcrowding of wells by duckweed fronds. At the end of the experiment, we photographed plates a second time (see Fig. S2 for representative images of initial and final plates). For all images, we used ImageJ (79) to calculate the total surface area occupied by *Lemna minor*. After the experiment, we ran 10 µL of well fluid through our NovoCyte Flow Cytometer to determine the final concentration of bacterial cells in wells. We measured the cell density (absolute cell count) and reduced noise by filtering out particulates below a certain size. We thresholded our samples based on forward and side scatter (threshold of 5,000) to exclude fragments of dead cells and plant matter. While we did not use fluorescent stains to distinguish live from dead bacterial cells, we thresholded our samples conservatively to reduce noise, and our estimates, which will include live and dead bacterial cells (80, 81), are likely a good proxy of bacterial fitness or community size.

### Data analysis

We fit statistical models in R version 4.3.1 (82). We modeled microbial cell density and duckweed growth in two ways. First, we tested whether plant or microbial productivity (change in duckweed biomass and microbial cell density) with a 10-strain community exceeded plant or microbial productivity in the average single-strain treatment, akin to a positive biodiversity-ecosystem function relationship. We fit linear mixed models using the “lme4” and “lmerTest” packages (83, 84), including the well location (edge or interior), plate number, and the identity of the bacterial strain nested within population as random effects. The plant growth model fit the final duckweed area (in pixels) as a function of the fixed effects of the initial duckweed area (in pixels) and treatment (three levels: control, single-strain inoculation, or 10-strain inoculation). For the plant growth model, we then used the “emmeans” package (85) for pairwise comparisons among treatment means using the Tukey method. For the microbial growth model, we subset the data to only wells that received microbes and fit final microbial cell density (log transformed to improve the normality of residuals) as a function of the fixed effects of treatment (two levels: single-strain inoculation or 10-strain inoculation), plant presence, and the interaction between treatment and plant presence.

We also wanted to compare the effect of inoculating with a 10-strain synthetic community to the additive expectation from the single-strain effects to ask whether microbes have sub-additive or synergistic effects on plant and microbial productivity (30). For example, when the productivity of the 10-strain community is less than the sum of the cell densities of the 10 strains grown alone, the results suggest more microbe-microbe competition than mutualism (4). For the microbes, we modeled the cell density of each population separately, with bacterial treatment (11 levels: the 10 single strains and the 10-strain community), plant presence, and the interaction between bacterial treatment and plant presence as fixed effects and well location (edge/interior) and plate number as random effects. For plant growth, we modeled the final duckweed area (in pixels) of each population separately, with the initial duckweed area (in pixels) and all 12 bacterial treatments (the 10 single strains, the 10-strain community, and control, uninoculated plants) as fixed effects, again including well location (edge/interior) and plate number as random effects. We considered the 10-strain synthetic community effect to be significantly different from the additive expectation from single-strain inoculations if the 95% confidence interval (CI) of the estimated marginal mean for the 10-strain community did not overlap the sum of the model coefficients for the 10 single strains (plus the control, in the case of plant growth only). We calculated estimated marginal means and additive expectations on the raw scale to facilitate comparisons and interpretation.

Finally, to examine fitness correlations between hosts and microbes, we again used the “emmeans” package (85) to extract the bacterial treatment means from linear mixed effects models for duckweed growth and microbial cell density. These models were fit on data standardized to a mean of 0 and standard deviation of 1 within each population and again included the random effects of well location and plate number. Our growth models also included the standardized initial pixel count as a fixed effect. As the plants used in our experiments are all clones and each of our bacterial treatments consisted of inocula developed from a single colony, variation within bacterial treatments is environmental, while variation in both microbe and host fitness among bacterial treatments is due to genetic differences among bacteria. We fit a simple linear regression between bacterial treatment means for duckweed and microbial growth to determine whether the fitness interests of microbes and duckweeds are aligned in each population.

## RESULTS

### Bacterial strain diversity

**Fig 2:**
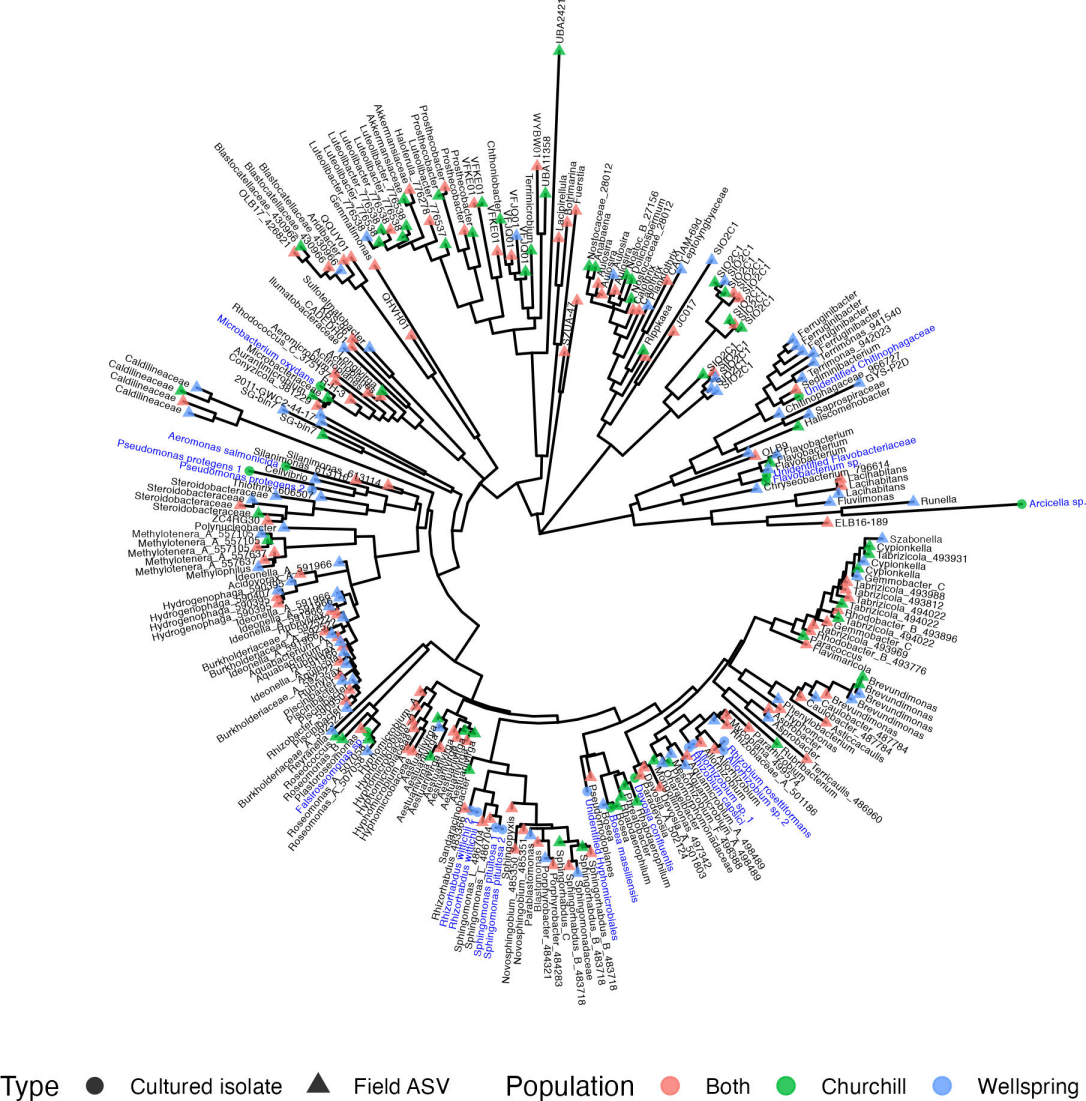
Phylogenetic tree of bacterial ASVs (triangles, tips labeled in black text with genus name when available, or family name) and single-strain isolates (circles, tips labeled in blue text) found at Churchill Marsh (green symbols), Wellspring Pond (blue symbols), or both populations (red symbols) reconstructed from 16S rRNA sequences.

We isolated a diversity of bacteria strains, with only *Pseudomonas protegens* shared between Churchill and Wellspring isolates (Fig. 2; Fig. S1; Tables S1 and S2). Bacteria isolated from Churchill duckweeds also included other Proteobacteria in the families Aeromonadaceae (*Aeromonas salmonicida*), Acetobacteraceae (*Falsiroseomonas* sp.), Boseaceae (or Beijerinckiaceae in the Greengenes2 taxonomy; see Fig. 2; Fig. S1) (*Bosea massiliensis*), and Devosiaceae (*Devosia confluentis*), as well as Bacteroidetes in the families Chitinophagaceae (an unidentified strain), Flavobacteriaceae (one *Flavobacterium* sp. and an unidentified strain), and Spirosomaceae (*Arcicella* sp.) and finally Actinobacteria in the family Microbacteriaceae (*Microbacterium oxydans*) (Fig. 2; Fig. S1). Isolates from Wellspring duckweeds were all Proteobacteria, including strains in the Sphingomonadaceae (two isolates each of *Sphingomonas pituitosa* and *Rhizorhabdus wittichii*), Rhizobiaceae (*Rhizobium* sp., *Rhizobium rosettiformans*, and two strains of *Allorhizobium*), and an unidentified *Hyphomicrobiales* isolate (Fig. 2; Fig. S1).

Cultured isolates were often closely related or identical to ASVs from field-collected duckweeds. 16S amplicon sequencing of field samples from Churchill Marsh and Wellspring Pond generated 98,403 and 87,809 raw reads, of which 22,445 and 19,541 remained, respectively, after all processing and filtering steps. These reads belonged to 170 ASVs in Churchill duckweeds and 184 ASVs in Wellspring duckweeds, with 110 ASVs shared between populations (Fig. 2; Fig. S1). Five of our 20 single-strain isolates exactly matched (i.e., 100% sequence identity) a field ASV, another 4 matched a field ASV at over 98% sequence identity, and 3 more matched a field ASV at over 95% sequence identity (Tables S1 and S2). Phylogenetic reconstruction showed that the 20 cultured isolates represented many of the major clades in the duckweed microbiome (Fig. 2).

### Microbial productivity

**Fig 3:**
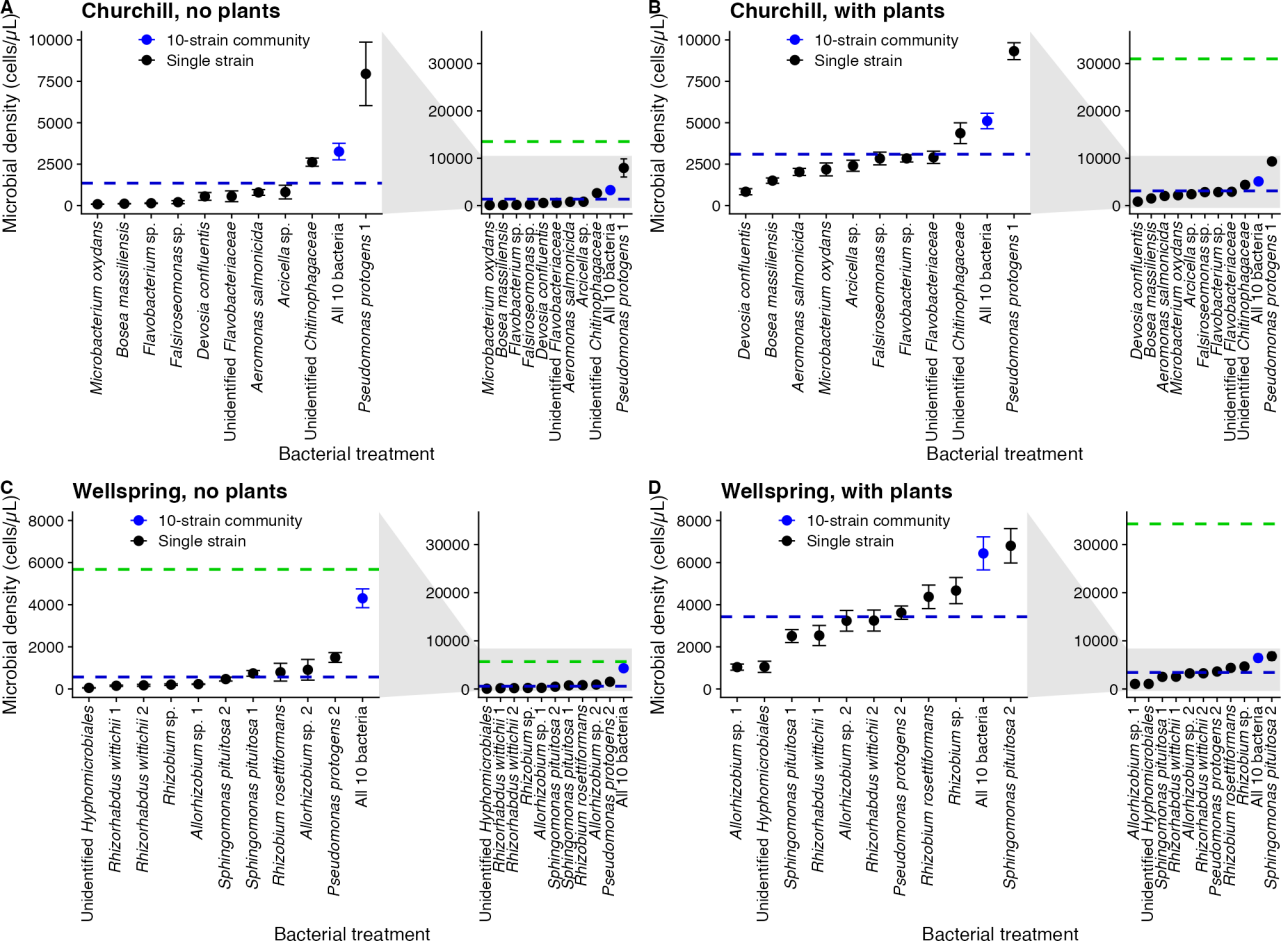
Microbial cell density (±1 SE) of bacterial strains and 10-strain synthetic communities in the absence (**A, C**) and presence (**B, D**) of *Lemna minor* from Churchill (**A, B**) and Wellspring (**C, D**). The blue dashed line is the mean of the single-strain effects. The green dashed line is the additive expectation for the 10-strain community. Each panel (**A–D**) shows the data plotted on an expanded *y*-axis scale that accommodates the additive expectation (on the right) and also zooms in on a smaller *y*-axis scale to help visualize differences among bacterial treatments (on the left). See also Tables S3 and S4.

**Fig 4:**
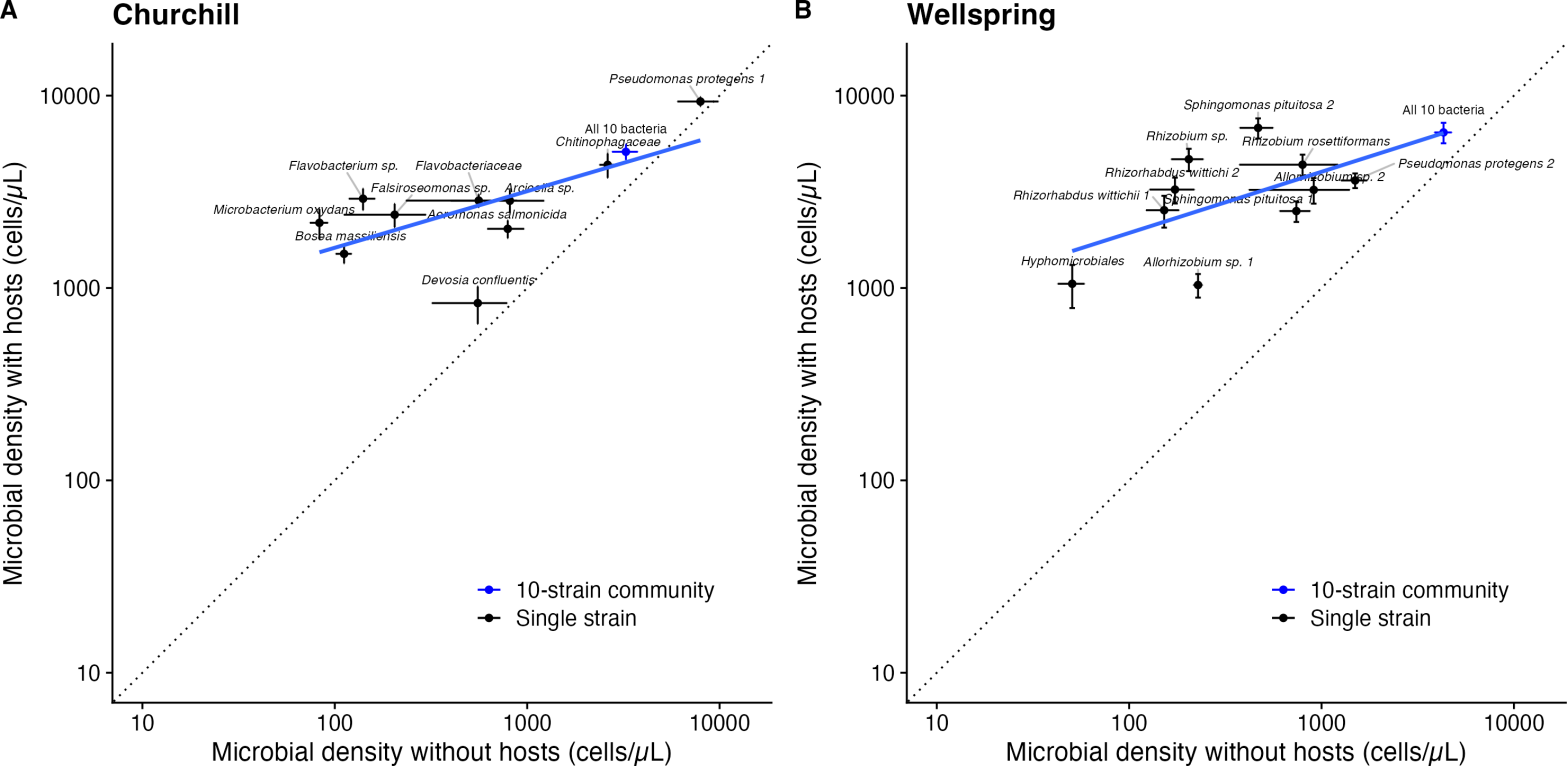
Microbial cell density (±1 SE) of bacterial strains (black dots) and 10-strain synthetic communities (blue dots) in the absence (*x*-axis) and presence (*y*-axis) of host plants from Churchill (**A**) and Wellspring (**B**). Points along 1:1 line (dotted) would indicate microbes or microbial communities that grew equally well with and without a host. Blue solid lines are simple linear regressions.

There was a significant biodiversity effect on microbial productivity. Ten-strain microbial communities were significantly more productive than the average single microbial strain (Table 1; Fig. 3), despite starting the experiment at the same cell density. Microbes also grew to significantly higher cell densities in the presence than in the absence of a plant host (Table 1), with slower-growing microbes (i.e., the microbes that grew to lower densities during the 10-day experiment) benefiting most from host presence (Fig. 4). Microbial growth without a host significantly predicted microbial growth with a host (Churchill: adjusted *R*^2^ = 0.409, *P* = 0.020; Wellspring: adjusted *R*^2^ = 0.335, *P* = 0.036), but strain and community means were always above the 1:1 line (dotted in Fig. 4) that would indicate equal growth with and without hosts. Nonetheless, the benefits of microbial diversity and host presence were sub-additive; there was a significantly negative biodiversity × host presence interaction effect (Table 1). The magnitude of this effect indicates that host presence increased the productivity of single microbial strains more than host presence increased the productivity of 10-strain microbial communities (Table 1; Fig. 4). In the model in Table 1, there were also significant random effects of plate (*P* < 0.001) and bacterial strain (*P* < 0.001) nested within population, but not well location (edge vs. interior) (*P* = 0.417), on microbial productivity.

**TABLE 1:** Model results for the effect of microbial strain diversity (one versus ten strains) on microbial productivity^*a*^

	Estimate	SE	df	*t*-value	*P* value
Intercept	5.464	0.211	18.340	25.870	<0.001
Ten-strain community	2.612	0.626	22.373	4.170	<0.001
Host presence	2.164	0.101	404.398	21.398	<0.001
Ten-strain community × host presence	−1.626	0.367	397.776	−4.431	<0.001

^*a*^ Intercept is a single microbial strain growing in the absence of a host. Estimates are on a natural log scale.

Microbe-microbe interactions affected microbial productivity in both the presence and absence of hosts. We used estimated marginal means from linear mixed models to calculate the additive expectation for the productivity of 10-strain communities from the single-strain effects (Tables S3 and S4). Ten-strain synthetic communities grew faster than almost all of the single strains considered individually, but the productivity of the 10-strain communities was usually significantly less than the sum of the cell densities of the 10 strains grown separately (compare the blue points and green dashed lines in Fig. 3; see Tables S3, S4). In no case did the 10-strain community achieve a cell density higher than the sum of the single-strain abundances at the end of the experiment (Fig. 3). Instead, interactions among microbes in 10-strain communities usually reduced the total microbial growth, consistent with widespread competition. Ten-strain communities of Wellspring microbes grown with host plants and 10-strain communities of Churchill microbes grown both with and without plants achieved cell densities significantly beneath the additive expectation from the single-strain effects (Tables S3 and S4). Only Wellspring microbes grown in the absence of host plants had an additive expectation that fell within the 95% CI for the 10-strain community. Furthermore, the productivity of the 10-strain communities was farther from the additive expectation in the presence (Fig. 3B and D) than in the absence (Fig. 3A and C) of a host, contrary to our *a priori* expectation that there would be more positive microbe-microbe effects with than without hosts. Instead, the greater sub-additivity when hosts are present suggests greater competition among microbes in the host than in the free-living environment.

### Host growth

**Fig 5:**
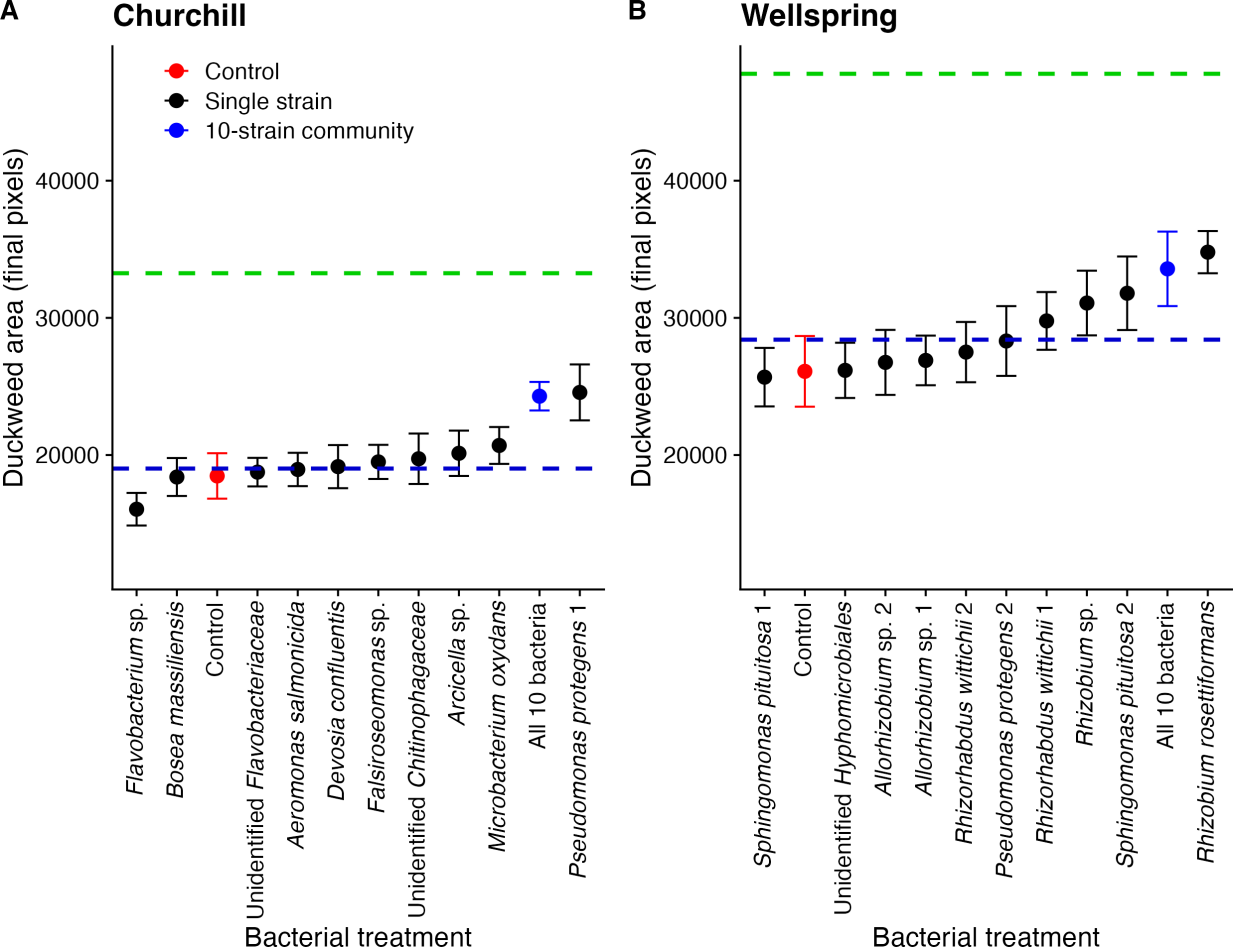
Growth (change in pixel area ± 1 SE) of *Lemna minor* from (A) Churchill Marsh and (B) Wellspring Pond in response to bacterial treatment. Red dots are uninoculated control plants, black dots are plants inoculated with a single strain of bacteria, and blue dots are plants inoculated with a 10-strain community. The blue dashed line is the mean of the single-strain effects. The green dashed line is the additive expectation for the 10-strain community. See also Tables S5 and S6.

There was a significantly positive microbial diversity effect on host growth. Ten-strain microbial communities significantly increased host growth more than the no-microbe control treatment (Table 2, Tukey’s post hoc test: *P* = 0.042) and more than the average single microbial strain (Tukey’s post hoc test: *P* = 0.036; Fig. 5). The random effects of edge (*P* < 0.001), plate number (*P* < 0.001), and population (*P* < 0.001), but not bacterial strain nested in population (*P* = 0.794), were also significant in the mixed model shown in Table 2. In contrast, although single bacterial strains had effects on duckweed growth that ranged from weakly negative to positive in Churchill and from neutral to positive in Wellspring (Fig. 5), the average single strain did not change the plant growth rate relative to the uninoculated control (Table 2, Tukey’s post hoc test: *P* = 0.682). Of the 20 single strains we tested, only *Pseudomonas protegens* significantly increased the growth of Churchill duckweeds compared with the uninoculated control (linear mixed model: *P. protegens*, *P* = 0.007, and all other strains, *P* > 0.05).

**TABLE 2:** Model results for the effect of microbial strain diversity (0, 1, or 10 strains) on plant growth^*a*^

	Estimate	SE	df	*t* value	*P* value
Intercept	22,258.34	4,725.52	1.86	4.710	0.049
Initial frond area (scaled)	6,756.27	433.57	456.84	15.583	<0.001
Single strain	1,362.99	1,614.19	20.01	0.844	0.408
Ten-strain community	5,752.16	2,203.46	20.99	2.611	0.016

^*a*^ Intercept is final frond area (in pixels) in the uninoculated control treatment, given mean initial frond area. Initial frond area was centered by subtracting the mean and scaled by dividing by the standard deviation.

In both populations, the 10-strain community effect was sub-additive and less than the sum of the effects of individual strains on duckweed growth (Fig. 5). However, the additive expectation was significantly greater than the effect of the 10-strain community only in Wellspring and not Churchill (Tables S5 and S6); in Churchill, the additive expectation fell just within the upper bound of the 95% CI for the effect of the 10-strain community (Table S5).

### Host-microbe fitness correlations

**Fig 6:**
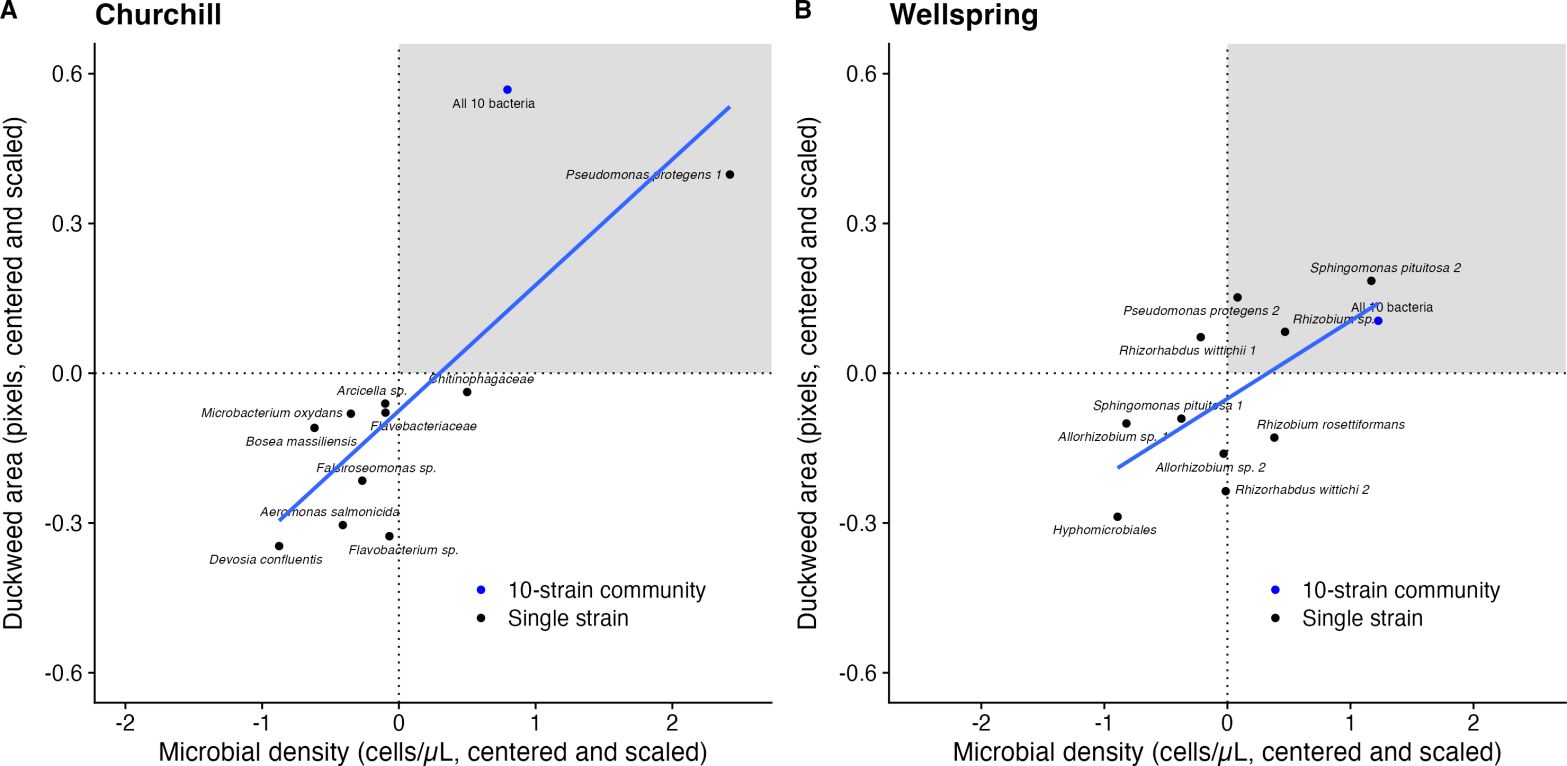
Fitness alignment between bacteria and their *Lemna minor* hosts for (A) Churchill Marsh and (B) Wellspring Pond. Plotted are estimated marginal means for each single-strain or 10-strain bacterial treatment for duckweed growth and microbial density. The gray-shaded regions show strains or communities that achieved above-average cell densities and above-average host growth.

Duckweed and bacterial fitness were significantly positively correlated in both populations (Churchill: 0.252 ± SE of 0.066, *P* = 0.004; Wellspring: 0.156 ± 0.058, *P* = 0.026; Fig. 6). No bacterial strains unambiguously benefitted at their hosts’ expense, as would be expected for pathogens.

## DISCUSSION

Plant microbiomes are complex communities of interacting microbes, but how interactions among microbes affect the outcome of host-microbiome interactions remains poorly understood. We isolated bacteria that represent many of the dominant taxa in the core duckweed microbiome (Fig. 2; Fig. S1) from field-collected *L. minor* (see also references 52, 57–60). Many of the cultured isolates were closely related or identical to the ASVs found on field-sampled duckweeds from the same sites; because we used different plants (from the same population) to culture bacteria and sequence field microbiomes, we would not expect perfect correspondence. After isolating bacteria, we tested the effects of single strains and 10-strain communities on microbial and host growth. Plant presence sharply increased microbial growth, but most single strains were commensals when tested individually, with only *Pseudomonas protegens* increasing the growth of *L. minor* from Churchill relative to uninoculated controls. However, 10-strain microbial communities led to greater plant growth compared with uninoculated controls, and both microbial and plant productivity were significantly greater with 10-strain communities than in the average single-strain treatment. We found that community effects were generally sub-additive, suggesting that emergent effects of microbiome diversity on microbial and host growth are mostly mediated by competition rather than facilitation among strains. We also tested whether host and microbe fitness interests are aligned and found that the microbes that reached the highest cell densities also provided the greatest benefits to plants.

### Microbial productivity

Microbes grew faster in the presence than the absence of plant hosts, something that is rarely tested in host-microbe experiments. In many systems, the environmental conditions a microbe encounters when free living are either unknown or hard to recreate in the lab. The microbes that associate with duckweeds, however, live in pond water and thus are readily cultured in minimal media, facilitating direct comparisons of their free-living and host-associated growth rates. Growth in the free-living environment significantly predicted growth with hosts, but not all microbes benefited equally from host presence. The rank order of bacterial productivity sometimes shifted between the host and no-host environment, especially in Wellspring where *Sphingomonas pituitosa* 2 had intermediate productivity in the absence of plants but was the fastest-growing single strain in the presence of hosts (Fig. 3). Furthermore, the strains that grew to the lowest cell densities without a host benefited most from host presence (Fig. 4), suggesting their growth is strongly limited by carbon exudates or other host-derived resources.

We found that 10-strain microbial communities were more productive than the average single strain but effects were always less than the sum of the single-strain cell densities. These results are in line with previous research on BEF and especially diversity-productivity relationships, including results from microbial communities (4, 13, 21, 22). More diverse communities may simply be more likely to contain “keystone” microbes (such as *P. protegens* in our Churchill population; Fig. 3A and B), or diversity-productivity relationships may emerge from niche complementary or facilitation among taxa (9, 23, 28). Our results also match previous findings of mostly sub-additive effects when microbes are co-cultured compared with when they are grown alone (references 1, 4 and references therein).

Foster and Bell (4) argued that simple Lotka-Volterra models predict that if two bacterial species compete, the sum of their abundances at equilibrium will be less when grown together than when grown alone. Thus, sub-additive effects such as those we observed should be evidence of at least some competition among microbes in mixed communities. Other studies have also employed this framework (e.g., reference 86). According to Foster and Bell, this logic should hold even in substitutive experimental designs such as ours, in which initial cell density was held constant regardless of the number of strains, such that each strain began at 1/10th the cell density in the 10-strain community as in the single-strain treatments. For any given taxon, there were initially 10 times more cells in the single-strain treatment than in the 10-strain treatment, but this is unlikely to bias results if microbial growth reached an equilibrial, stationary phase in all treatments (because the population size at equilibrium does not depend on initial densities). We did not measure whether microbes had attained a stationary phase, which would have required removing plate seals and risking contamination, but 10 days is more than sufficient for most bacteria to reach their carrying capacities under similar growth conditions as ours. Our results therefore suggest that at least some microbes in our 10-strain communities compete for resources.

Niche overlap is common among microbes, resulting in competition for resources or space. A study of endophytic bacterial communities of the prairie grass *Andropogon gerardii*, for example, found that pairwise combinations of bacteria exhibit a mean niche overlap of roughly 60% (87). Network and metagenomic analyses of microbial communities (88, 89) have also underscored the high niche or metabolic overlap among microbes in many microbiomes. Niche overlap is especially likely when microbes are isolated under the same culture conditions, as we did here, and we might have found less competition among strains by isolating microbes on different types of media. However, even using only a single media type, the bacteria we isolated were from diverse clades from across the natural microbiome of field-sampled duckweeds (Fig. 2), suggesting that our 10-strain synthetic communities still captured some of the phylogenetic and metabolic diversity of wild duckweed microbiomes.

Interactions among microbes in a community are often a mix of competitive and facilitative (90), sometimes even within the same pairwise interaction (91). Members of microbial communities can facilitate one another’s growth despite widespread competition for resources (28, 90, 91). Many kinds of mutually beneficial exchanges occur between microbes, such as cross-feeding or complementary ecosystem services such as nitrogen fixation or biofilm formation (2, 25, 29, 92, 93). For two strains, we can infer that competition is stronger than any mutualistic exchange of metabolites or other services if microbial productivity is less when the two strains are grown together than the sum of their final cell densities when grown apart. However, when comparing the growth of 10 strains in a community to 10 single strains, it is challenging to determine how many interactions are competitive versus facilitative. When microbial productivity in a species-rich community does not exceed the sum of the growth of each component microbe in isolation, this merely tells us that competition among microbes is not fully compensated by any microbe-microbe mutualisms that are present; it does not mean facilitation does not occur in the microbiome. Indeed, in the context of limited resources, as the number of species in the community increases, it rapidly becomes impossible for microbial productivity to exceed the sum of individual species effects, as microbial communities reach the maximum density afforded by resources in their environments (e.g., reference 23).

We expected to find that hosts increased positive interactions between microbes, because if one microbe increases host growth, it may indirectly benefit another microbe. In our experiments, duckweeds were the only source of fixed carbon available to bacteria, which should result in greater positive interactions among the multiple mutualists of a focal host species (30, 31). However, if anything, the productivity of 10-strain communities was closer to the additive expectation from single-strain treatments in the absence than in the presence of plants, suggesting greater facilitation without than with hosts (Fig. 3). While this result conflicts with what we expected, it may highlight the ecological contingency of bacterial cooperation on the nutrient environment. Several studies (e.g., references 28, 94) have demonstrated that bacterial facilitation is more likely under stressful, nutrient-poor conditions; in our experiments, *L. minor* fronds substantially increased the availability of nutrients in wells through the production of root exudates (32, 33), potentially resulting in the emergence of more competitive interactions among strains.

### Host growth

Although single microbial strains benefited from the presence of duckweed hosts (Fig. 3 and 4), the effect was rarely reciprocal; most single strains had no effect on plant growth, making them commensals when tested in isolation (Fig. 5). The only exception was *P. protegens*, which increased the growth of Churchill *L. minor* on its own. *Pseudomonas protegens* is a widespread and often plant growth-promoting bacterium (e.g., references 95, 96). However, despite mostly neutral effects of single strains on plant growth, 10-strain synthetic communities significantly increased *L. minor* growth rates, in keeping with other work on the mutualistic effects of microbiomes on duckweeds (19, 24, 52, 58; but see reference 97). Thus, beneficial microbiomes can be assembled from strains that are largely commensals in isolation, suggesting that host-microbiome mutualisms are an emergent property of interactions among microbial strains.

The competition among bacteria in 10-strain communities indicated by the microbial productivity results could have either increased or decreased the net benefits microbiomes provide to hosts, depending on whether competitively dominant strains are also highly beneficial to hosts and on whether the strains they suppress are mainly beneficial or pathogenic for hosts (9, 11, 12, 30). Similarly, whether any positive interactions among microbes increase or decrease host benefits depends on whether these interactions promote the growth of microbes that help or harm hosts. Thus, how interactions among microbes affect microbial productivity may not match their effects on host growth.

Nonetheless, we found a similar pattern for 10-strain versus single-strain effects on host growth as for microbial productivity. Inoculation with a 10-strain synthetic community increased host growth more than inoculation with the average single bacterial strain but did not exceed the benefits of the best-performing single strain. The community inoculation effects we observed may simply reflect the sampling effect, with more diverse communities having higher microbial productivity and host benefits because they are more likely to contain keystone microbes, such as *P. protegens* in Churchill, that exert disproportionate effects on microbial and duckweed growth (9, 12, 13, 18, 19, 98). These results also have implications for microbiome applications in agriculture or medicine; complex communities may not outperform the most beneficial microbial strains.

### Fitness alignment

Outside legume-rhizobium interactions, few studies have measured fitness alignment or conflict between plants and their microbes (but see references 52, 61). As in the study by O’Brien et al. (52), we found that host and microbe fitness was positively correlated in both study populations (Fig. 6), although fitness alignment was stronger in Churchill Marsh than in Wellspring Pond. No individual strains significantly increased their own fitness above the population average while reducing host fitness below its population average (Fig. 6), as we would expect for strong pathogens or “cheaters” (99). The legume-rhizobium literature has also found little evidence of cheaters (49; but see reference 100). Instead, ineffective rhizobia appear to be regularly out-competed and replaced by more beneficial strains (51). That host and microbe fitness interests are closely aligned even in the more facultative associations between duckweeds and microbes is somewhat surprising, given that we did not pre-select only beneficial microbial strains to use in our experiments and we would have expected to sample some plant pathogens simply by chance.

Compared with the legume-rhizobium symbiosis, plant microbiomes involve much greater bacterial diversity (101), and associations between hosts and particular microbes are less reliable across time and space (102). Duckweed microbiomes, and plant microbiomes more broadly, are largely environmentally acquired, and while host genotypes can control aspects of microbiome community assembly, such processes often act with low resolution on broad phylogenetic differences among microbes (41, 57, 103, 104), rather than on the strain-level variation that often mediates microbial effects on plants (12, 18, 19). Nonetheless, our results suggest that duckweed-microbe fitness interests may be aligned “by default,” just as in legume-rhizobium interactions with little coevolutionary history (49). As such, plant-microbiome interactions may not require the evolution of specific host control mechanisms (105, 106) to maintain mutualism between partners.

## Data Availability

All phenotype data and R and QIIME 2 scripts supporting this manuscript are publicly available at https://github.com/JasonLaurich/Lemna_single_inoculations. Single-strain 16S rRNA Sanger sequences have been deposited at GenBank (accession numbers in Tables S1 and S2), and field microbiome 16S rRNA sequences have been deposited at the NCBI Sequence Read Archive (BioProject accession PRJNA1098713).

## SUPPLEMENTAL MATERIAL

Supplemental material mbio.00972-24-s0001.pdf Supplemental figures and tables.

ASM does not own the copyrights to Supplemental Material that may be linked to, or accessed through, an article. The authors have granted ASM a non-exclusive, world-wide license to publish the Supplemental Material files. Please contact the corresponding author directly for reuse.
