# Supplementary material for: Community interactions among microbes give rise to host-microbiome mutualisms in an aquatic plant
Source: mBio. 2024 Jun 21;15(7):e00972-24. doi: 10.1128/mbio.00972-24 (PMC11324027; doi:10.1128/mbio.00972-24)
Supplement: Supplemental material — Supplemental figures and tables. [file mbio.00972-24-s0001.pdf]

# Supplemental Material

**Table S1.** 16S rRNA sequence GenBank accession numbers and taxonomic identifications for single microbial strains isolated from duckweeds collected from Churchill Marsh.

| | Closest NCBI match | | Closest ASV in field data | | This study |
|---|---|---|---|---|---|
| GenBank accession | Taxonomic name | Identity (%) | Taxonomic name | Identity (%) | Taxonomic name |
| PP504234 | *Aeromonas salmonicida* | 99.93 | *Cellvibrio* | 86.75 | *Aeromonas salmonicida* |
| PP504232 | *Arcicella aurantiaca* | 97.65 | *Runella palustris* | 78.48 | *Arcicella* sp. |
| PP504226 | *Bosea massiliensis* | 98.56 | *Bosea caraganae* | 100.00 | *Bosea massiliensis* |
| PP504231 | *Devosia confluentis* | 98.18 | *Paradevosia shaoguanensis* | 100.00 | *Devosia confluentis* |
| PP504228 | *Falsiroseomonas stagni* | 96.78 | *Roseomonas* A_507058 *stagni* | 99.21 | *Falsiroseomonas* sp. |
| PP504229 | *Flavobacterium succinicans* | 95.70 | *Flavobacterium dankookense* | 92.88 | *Flavobacterium* sp. |
| PP504225 | *Flavobacterium succinicans* | 93.85 | *Flavobacterium dankookense* | 92.50 | Unidentified *Flavobacteriaceae* |
| PP504233 | *Microbacterium oxydans* | 99.16 | *Microbacteriaceae* | 96.51 | *Microbacterium oxydans* |
| PP504227 | *Parasediminibacterium paludis* | 94.10 | *Sediminibacterium* sp. | 92.75 | Unidentified *Chitinophagaceae* |
| PP504230 | *Pseudomonas protegens* | 100.00 | *Cellvibrio* | 86.07 | *Pseudomonas protegens* 1 |

**Table S2.** 16S rRNA sequence GenBank accession numbers and taxonomic identifications for single microbial strains from Wellspring Pond.

| | Closest NCBI match | | Closest ASV in field data | | This study |
|---|---|---|---|---|---|
| GenBank accession | Taxonomic name | Identity (%) | Taxonomic name | Identity (%) | Taxonomic name |
| PP515680 | *Flaviflagellibacter deserti* | 91.83 | *Pseudorhodoplanes sinuspersici* | 93.81 | Unidentified *Hyphomicrobiales* |
| PP515685 | *Pseudomonas protegens* | 100.00 | *Cellvibrio* | 88.50 | *Pseudomonas protegens* 2 |
| PP515687 | *Rhizobium capsici* | 97.32 | *Allorhizobium ipomoeae* | 96.00 | *Rhizobium* sp. |
| PP515688 | *Rhizobium capsici* | 93.25 | *Allorhizobium ipomoeae* | 96.00 | *Allorhizobium* sp. 1 |
| PP515682 | *Rhizobium rosettiformans* | 99.02 | *Allorhizobium wuzhouense* | 100.00 | *Rhizobium rosettiformans* |
| PP515683 | *Rhizobium rosettiformans* | 92.54 | *Allorhizobium wuzhouense* | 99.00 | *Allorhizobium* sp. 2 |
| PP515686 | *Rhizorhabdus wittichii* | 98.59 | *Rhizorhabdus* 483366 | 100.00 | *Rhizorhabdus wittichi* 1 |
| PP515681 | *Rhizorhabdus wittichii* | 98.52 | *Rhizorhabdus* 483366 | 100.00 | *Rhizorhabdus wittichi* 2 |
| PP515684 | *Sphingomonas pituitosa* | 99.22 | *Sphingomonas* L_486704 *spermidinifaciens* | 98.03 | *Sphingomonas pituitosa* 1 |
| PP515689 | *Sphingomonas pituitosa* | 98.91 | *Sphingomonas* L_486704 *spermidinifaciens* | 98.03 | *Sphingomonas pituitosa* 2 |

**Table S3.** Estimated marginal means and 95% confidence intervals (CIs) for the productivity of each single microbial strain and the 10-strain synthetic community from Churchill in the absence (left-hand columns) and presence (right-hand columns) of a host. Estimates are in units of cells/$\mu$L.

| | No host | | | Host | | |
|---|---|---|---|---|---|---|
| Treatment | Estimate | 95% CI lower | 95% CI upper | Estimate | 95% CI lower | 95% CI upper |
| *Aeromonas salmonicida* | 687.39 | -674.01 | 2048.78 | 1996.78 | 808.59 | 3184.97 |
| *Arcicella* sp. | 850.24 | -456.00 | 2156.48 | 2757.73 | 1323.88 | 4191.58 |
| *Bosea massiliensis* | 172.96 | -1408.67 | 1754.60 | 1490.98 | 196.35 | 2785.60 |
| *Devosia confluentis* | 471.16 | -964.17 | 1906.49 | 833.18 | -734.54 | 2400.90 |
| *Falsiroseomonas* sp. | 151.04 | -1134.22 | 1436.29 | 2378.78 | 1112.53 | 3645.02 |
| *Flavobacterium* sp. | 101.90 | -1261.05 | 1464.86 | 2839.56 | 1124.02 | 4555.10 |
| *Microbacterium oxydans* | 60.49 | -1264.82 | 1385.80 | 2212.05 | 730.65 | 3693.45 |
| *Pseudomonas protogens* 1 | 7907.95 | 6359.00 | 9456.89 | 9325.02 | 7757.22 | 10892.83 |
| Unidentified *Chitinophagaceae* | 2574.86 | 777.00 | 4372.73 | 4369.78 | 2787.22 | 5952.33 |
| Unidentified *Flavobacteriaceae* | 544.47 | -750.33 | 1839.27 | 2791.53 | 1307.78 | 4275.28 |
| 10-strain community | 3258.23 | 1805.23 | 4711.24 | 5086.94 | 3465.51 | 6708.36 |
| Additive expectation | 13522.45 | | | 30995.38 | | |

**Table S4.** Estimated marginal means and 95% confidence intervals (CIs) for the productivity of each single microbial strain and the 10-strain synthetic community from Wellspring in the absence (left-hand columns) and presence (right-hand columns) of a host. Estimates are in units of cells/$\mu$L.

| | No host | | | Host | | |
|---|---|---|---|---|---|---|
| Treatment | Estimate | 95% CI lower | 95% CI upper | Estimate | 95% CI lower | 95% CI upper |
| *Allorhizobium* sp. 1 | 172.77 | -1397.62 | 1743.15 | 1153.05 | -336.36 | 2642.46 |
| *Allorhizobium* sp. 2 | 982.80 | -534.05 | 2499.65 | 3454.32 | 1879.12 | 5029.53 |
| *Pseudomonas protogens* 2 | 1530.35 | -101.48 | 3162.18 | 3787.26 | 2286.56 | 5287.95 |
| *Rhizobium rosettiformans* | 950.69 | -569.59 | 2470.96 | 4420.19 | 2898.85 | 5941.53 |
| *Rhizobium* sp. | 139.64 | -1400.50 | 1679.78 | 4785.96 | 3297.26 | 6274.66 |
| *Rhizorhabdus wittichii* 2 | 91.46 | -1385.34 | 1568.26 | 3385.86 | 1838.66 | 4933.06 |
| *Rhizorhabdus wittichii* 1 | 191.66 | -1398.90 | 1782.23 | 2863.33 | 1277.93 | 4448.74 |
| *Sphingomonas pituitosa* 1 | 885.94 | -663.46 | 2435.33 | 2556.60 | 1044.40 | 4068.80 |
| *Sphingomonas pituitosa* 2 | 569.81 | -945.62 | 2085.24 | 6854.24 | 5360.65 | 8347.84 |
| Unidentified *Hyphomicrobiales* | 162.92 | -1394.17 | 1720.01 | 1030.22 | -590.03 | 2650.46 |
| 10-strain community | 4290.43 | 2799.37 | 5781.49 | 6785.64 | 4966.21 | 8605.08 |
| Additive expectation | 5678.03 | | | 34291.03 | | |

**Table S5.** Estimated marginal means and 95% confidence intervals (CIs) for host growth (final size) with each single microbial strain and the 10-strain synthetic community from Churchill. Estimates are in units of pixels.

| Treatment | Estimate | 95% CI lower | 95% CI upper |
|---|---|---|---|
| Uninoculated control | 17432.61 | 8614.11 | 26251.10 |
| *Aeromonas salmonicida* | 17249.67 | 8426.63 | 26072.71 |
| *Arcicella* sp. | 19691.11 | 10966.69 | 28415.52 |
| *Bosea massiliensis* | 19047.58 | 10136.60 | 27958.55 |
| *Devosia confluentis* | 16874.00 | 7964.69 | 25783.32 |
| *Falsiroseomonas* sp. | 18140.25 | 9229.93 | 27050.58 |
| *Flavobacterium* sp. | 17119.51 | 8406.84 | 25832.18 |
| *Microbacterium oxydans* | 19224.18 | 10312.96 | 28135.39 |
| *Pseudomonas protogens* 1 | 23856.68 | 15052.57 | 32660.79 |
| Unidentified *Chitinophagaceae* | 19698.31 | 10876.70 | 28519.91 |
| Unidentified *Flavobacteriaceae* | 19247.03 | 10383.66 | 28110.39 |
| 10-strain community | 25307.30 | 16652.41 | 33962.19 |
| Additive expectation | 33254.86 | | |

**Table S6.** Estimated marginal means and 95% confidence intervals (CIs) for host growth (final size) with each single microbial strain and the 10-strain synthetic community from Wellspring. Estimates are in units of pixels.

| Treatment | Estimate | 95% CI lower | 95% CI upper |
|---|---|---|---|
| Uninoculated control | 26257.86 | 18526.25 | 33989.47 |
| *Allorhizobium* sp. 1 | 27565.54 | 19795.61 | 35335.46 |
| *Allorhizobium* sp. 2 | 26917.00 | 19198.91 | 34635.09 |
| *Pseudomonas protogens* 2 | 31114.63 | 23390.79 | 38838.46 |
| *Rhizobium rosettiformans* | 27707.19 | 19922.92 | 35491.46 |
| *Rhizobium* sp. | 30311.58 | 22593.16 | 38030.01 |
| *Rhizorhabdus wittichii* 2 | 25676.23 | 17904.21 | 33448.25 |
| *Rhizorhabdus wittichii* 1 | 30005.09 | 22254.31 | 37755.87 |
| *Sphingomonas pituitosa* 1 | 27742.37 | 20036.76 | 35447.98 |
| *Sphingomonas pituitosa* 2 | 31894.35 | 24206.70 | 39582.01 |
| Unidentified *Hyphomicrobiales* | 25180.41 | 17466.41 | 32894.41 |
| 10-strain community | 30755.44 | 22986.11 | 38524.78 |
| Additive expectation | 47793.64 | | |

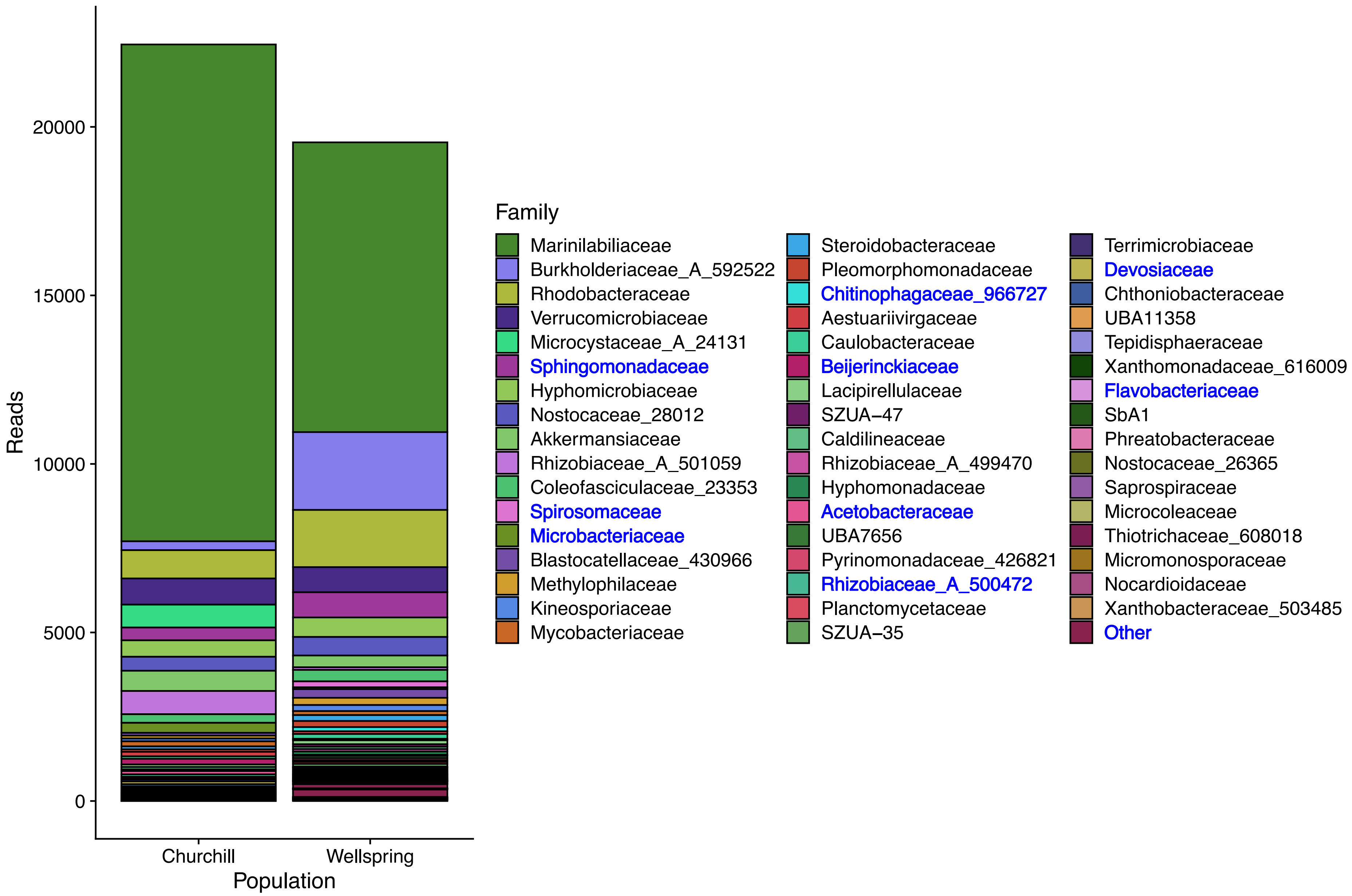


**Figure S1.** Read abundance of bacterial families in the microbiome of field-collected *Lemna minor* duckweeds from Churchill Marsh (left) and Wellspring Pond (right). Names in bolded blue font are families from which isolates were cultured and tested on *L. minor* in experiments. "Other" collapses families with less than 0.1% of reads.

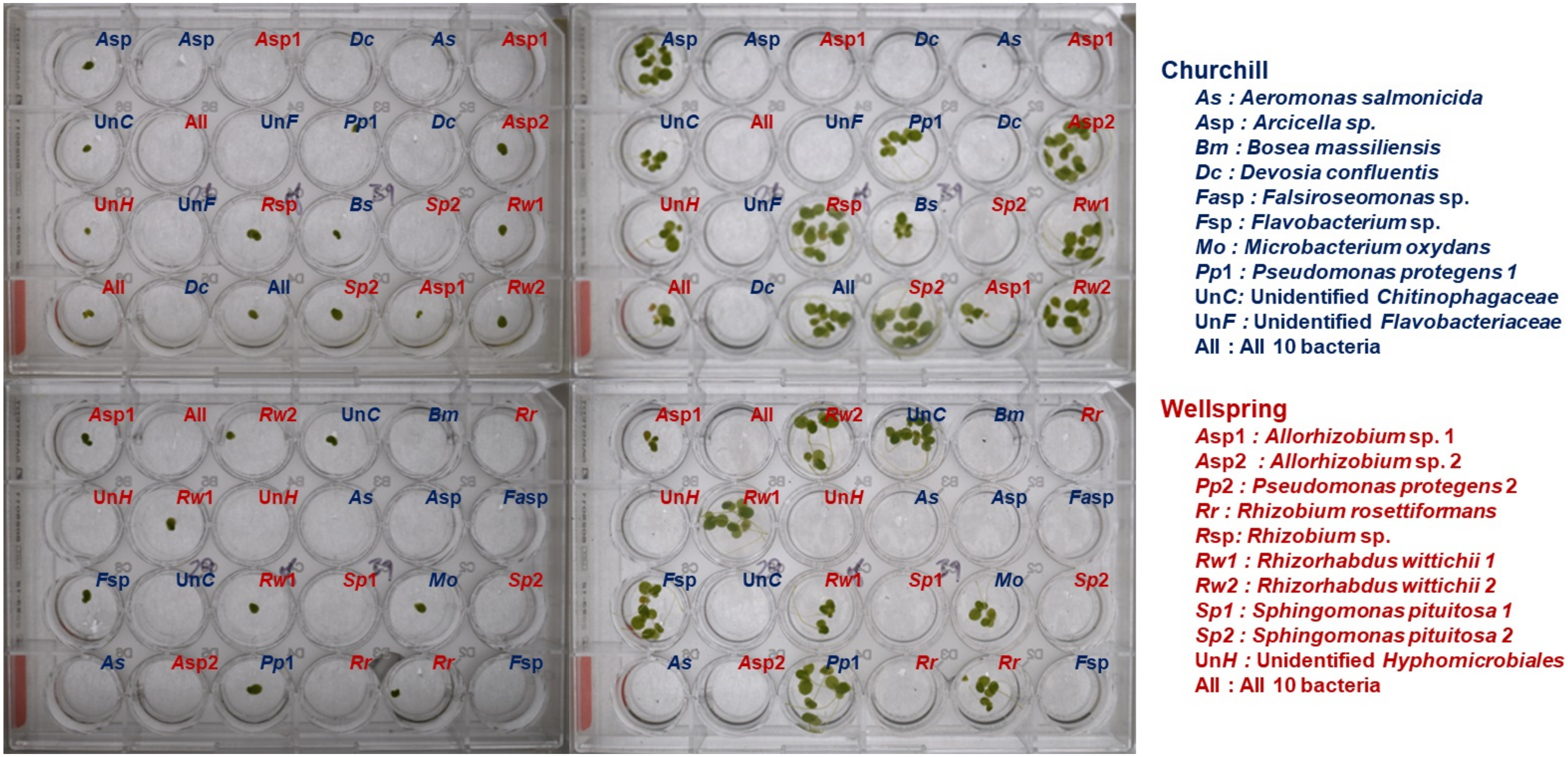


**Figure S2.** Representative plates showing the random assignment of *Lemna minor* fronds and bacterial treatments to wells for the Churchill (blue) and Wellspring (red) populations. Left-hand images were taken immediately after microbial inoculation and sealing; right-hand images are the same plates after 10 days of growth.
